# Reconsideration of the nomadic condition of the southernmost Guachichiles based on the relationship with their environment

**DOI:** 10.1186/s13002-018-0223-x

**Published:** 2018-04-02

**Authors:** Eric Mellink, Mónica E. Riojas-López, José Antonio Rivera-Villanueva

**Affiliations:** 1 Departamento de Biología de la Conservación, Centro de Investigación Científica y de Educación Superior de Ensenada, Ensenada, B.C. Mexico; 2 Departamento de Ecología, Centro Universitario de Ciencias Biológicas y Agropecuarias, Universidad de Guadalajara, Zapopan, Jalisco Mexico; 3 El Colegio de San Luis, San Luis Potosí, Mexico

**Keywords:** Chichimecas, Jalisco, Guanajuato, México, Tunal Grande

## Abstract

**Background:**

The Guachichiles were a group of Chichimeca people that inhabited the southern and central parts of the Mexican Plateau. In the southern area of their distribution, they occupied and used the *tunales*, extensive forests of arborescent *nopales* (*Opuntia* spp.). Their pre-Columbian distribution was dissected by the Royal Silver Road established by the Spaniards, and this lead to them being main protagonists in the so-called Chichimeca War, during the sixteenth century. With very little first-hand documentation, the Guachichiles were described as savage, warring, primitive, hunting nomads, but little efforts have been done to understand their daily life habits. Based on the relationship of pre-Columbian southern Guachichiles with their environment, we re-valuate whether they were nomads, as the Chichimecas collectively have been labeled, or whether those living in *tunales* could live year-round in this habitat. As part of our analysis, we propose the primary plant and animal species that integrated their diet.

**Methods:**

We draw information from a review of bibliographic sources, complemented with extensive searches in all pertinent Mexican archives. We carried out field work to define the geographical extent of the pre-Columbian territory of the southernmost Guachichiles, based on the Spanish Chronicles, remnant fragments of vegetation, landscape characteristics, and geographic names related with *nopales*. Using approaches from wildlife ecology, historical sciences and ethnobiological information on wild resources currently or recently used in the area, we proposed which resources were available to the southernmost Guachichiles, and how their primary diet might have been.

**Results:**

The habitat of the southern Guachichiles, the *tunal* forest, was exuberant and rich in resources, having provided numerous plant products, of which *tunas* (prickly pears) and mesquite pods were of uttermost importance. At least 10 plant foods were available within the *tunales.* They would have consumed at least seven birds (including their eggs), six mammals, four reptiles, grubs, and honey, in addition to at least six vertebrate species hunted at the edges of the *tunal* with grasslands and shrublands or in more open patches of *tunal*. In addition to food, they prepared at least three alcoholic beverages, had access to two species of probable psychoactive beehive cacti and to one hallucinogenic mushroom species, and might have traded peyote from the north with outside-*tunal* Guachichiles.

**Conclusions:**

The rich habitat in which southern Guachichiles lived allowed them to be largely sedentary, but this required that they prevented other groups from gathering and hunting in their habitat. As a result of them living in and defending the *tunales*, the Guachichiles could have been divided into two or three habitat-driven groups: *Tunal* Guachichiles, and grassland and, or shrubland Guachichiles.

## Background

“Poorly known groups of the Gulf Coastal Plain and Interior” was the denomination used by the authoritative Smithsonian Institution’s Handbook of North American Indians [1, 2] in reference to people that lived in the semiarid Mexican plateau, from northeastern Guanajuato and southern San Luis Potosí northwards, by the time of the Spanish arrival to the “new world.” This area, that supported one of the most unique habitats in North America, large expanses covered by forests of arborescent *nopales* (prickly pears, *Opuntia* spp.), was occupied mainly by the Guachichiles (also spelled Huachichiles and Cuachichiles), a people famed for their “savagery” but about which very little is known until present.

**Fig. 1:**
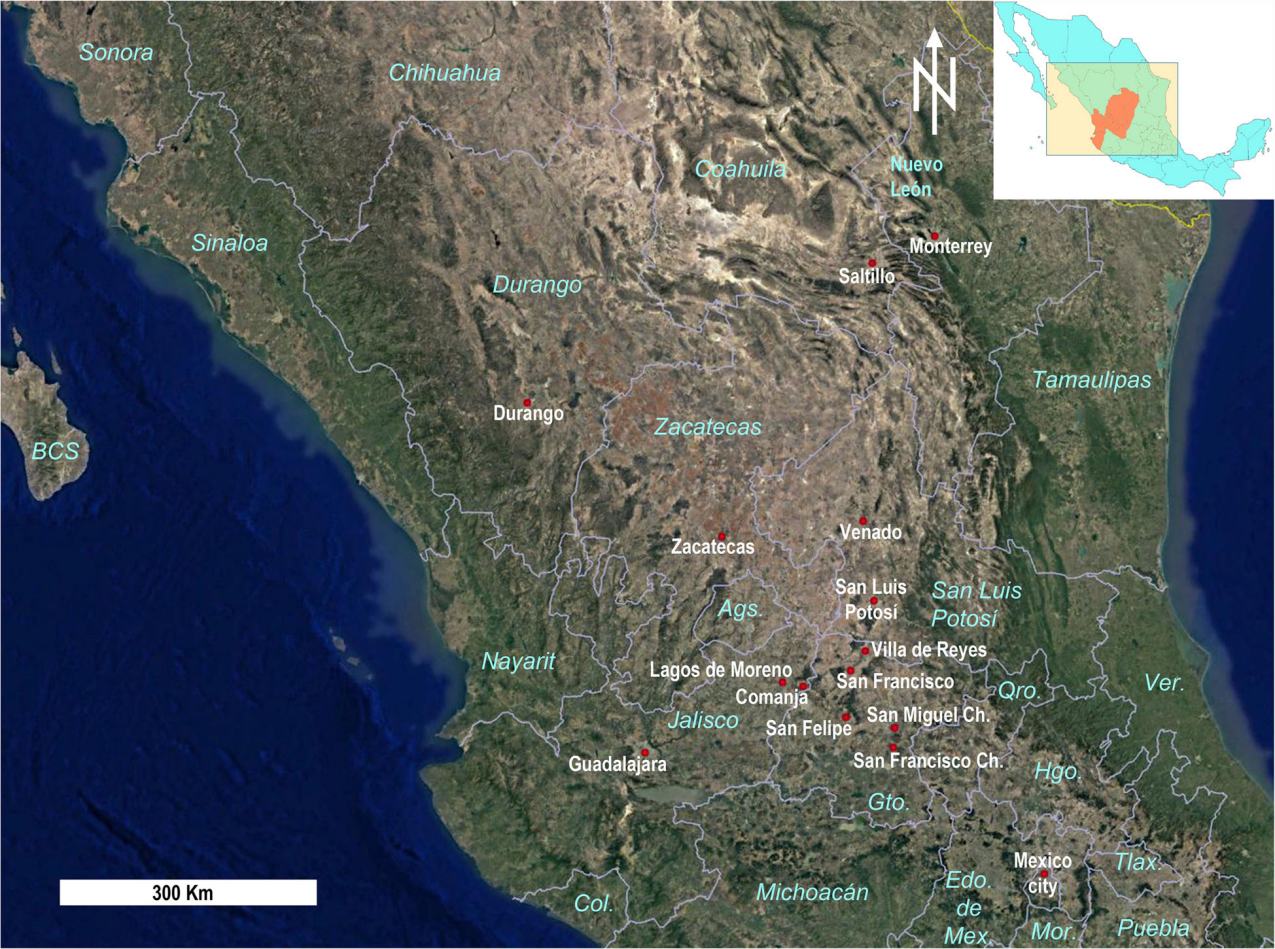
Villages mentioned in text, and current political division of central and northern Mexico. Villages are indicated in white, and states in blue italics. The orange area in the inlaid reference map corresponds to Spanish Kingdom of Nueva Galicia. State abbreviations are BCS = Baja California Sur, Ags. = Aguascalientes, Qro. = Querétaro, Ver. = Veracruz, Hgo. = Hidalgo, Gto. = Guanajuato, Col. = Colima, Edo. de Mex. = Estado de México, Tlax. = Tlaxcala, and Mor. = Morelos. Base map: Google Earth, ©2014 DigtalGlobe

When the Spaniards started exploring and finding silver mines in northern Mexico in the mid-sixteenth century, they found those lands occupied by hunting-gathering peoples [3]. The Spanish called them collectively Chichimecas. The term was ambiguous, as the Mexica had used it for naming previous people of the Valley of Mexico (site of current Mexico City) and other nearby groups which they considered primitive, as Fr. Bernardino de Sahagún described in his sixteenth century masterpiece [4] (see also Powell [5] for an excellent review of the use of the concept of Chichimeca). In the concept of the Spanish, as was referred to by Joaquín García Icazbaleta in the second edition (from 1877) of the González de Eslava’s 1610 religious theatre  plays [6], the name Chichimeca was used to name all non-reduced Indians of Western and Northern Mexico, including many groups and different languages [7]. As the Spanish knowledge of native people increased, the term Chichimeca was constrained to those groups on the central plateau, inhabiting the area being colonized, to about Saltillo and Durango (see Fig. 1 for all places named in the text).

### The Chichimecas

**Fig. 2:**
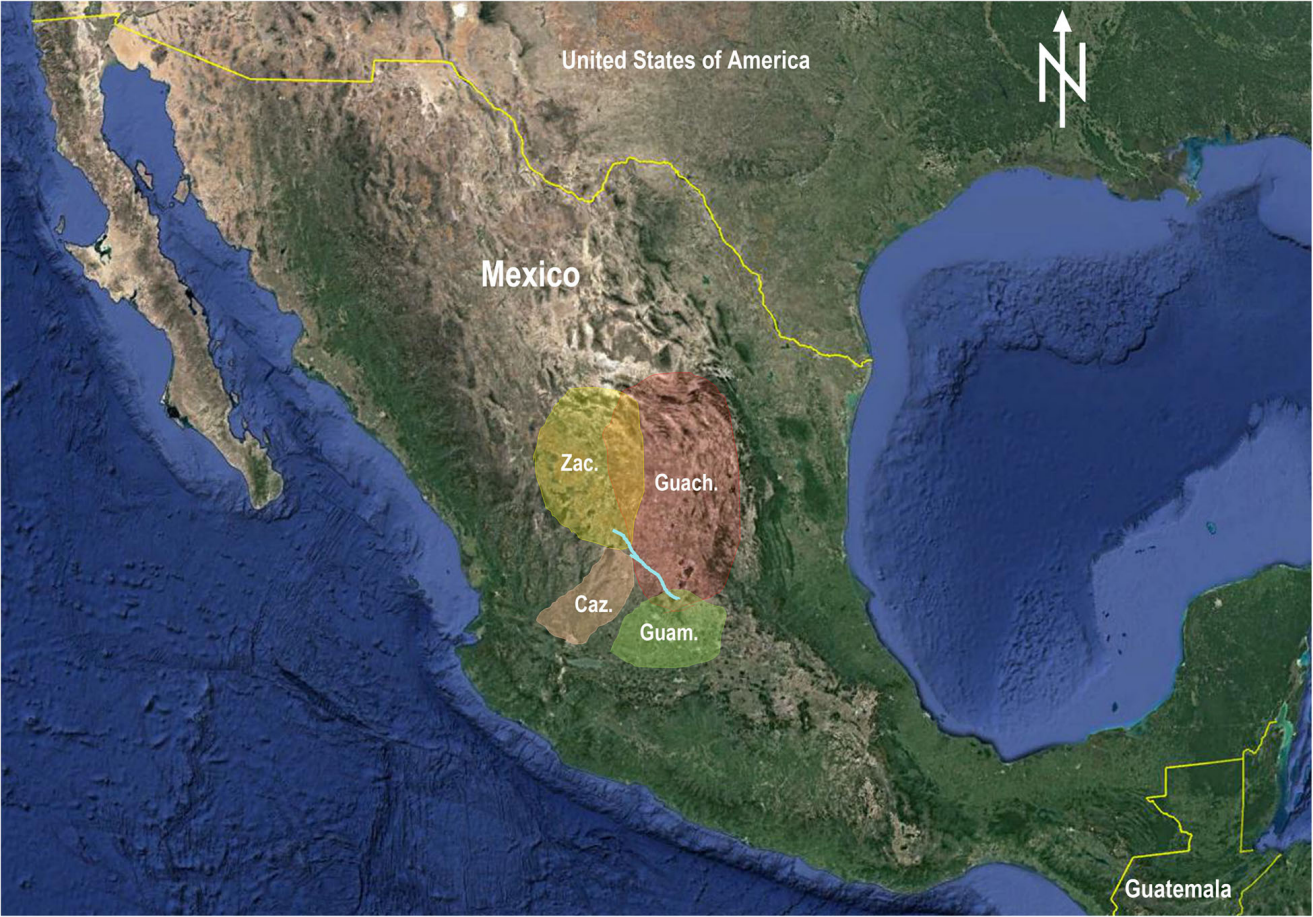
Approximate distribution of the major Chichimeca groups in the mid-sixteenth century. The blue line represents the section of the Camino Real de Tierra Adentro between the presidios of San Felipe and Zacatecas (currently cities). Abbreviations are: Zac. = Zacatecos, Guach. = Guachichiles, Caz. = Cazcanes, and Guam. = Guamares. Drawn after Powell 1945. Base map: Google Earth, ©2014 DigtalGlobe

According to the latter definition, the Chichimecas included four major distinct groups: Guachichiles, Zacatecos, Cazcanes, and Guamares (Fig. 2), in addition to a number of other smaller groups. Their relationships cannot be analyzed, as their culture and languages were lost long time ago [8], without leaving any vestiges except for a few names of their leaders (see, for example, [5]), of which some were adopted as geographic place names.

During the Spanish colonization, the word “chichimeca” became the epitome of savage, barbarian, and assassin. Fernán González de Eslava [6] describes this idea eloquently in his Coloquio Quinto (the fifth part of a series of spiritual and sacramental plays published originally in 1610):

**Table:** 

Dentro en su furor esquivo Se encierran todos los males, Y con flechas infernales A ninguno dejan vivo De los míseros mortales.	Within their elusive fury Are all evils enclosed And with infernal arrows They leave alive no one Of the wretched mortals
El Demonio, Carne y Mundo Son Chichimecos malditos, Que. nos espantan con gritos Que. nos llevan al profundo Con gravísimos delitos.	The Demon, Flesh and World Are damned Chichimecos That scare us with shoutings That bring us to the depth With most serious offenses

All writers at that time and later ones considered the Chichimecas barbarians that did not have settlements but roamed through the country, hunting; sleeping where the night found them [4, 7, 9, 10]. According to [7, 11, 12], the Chichimecas were tall, strong, and “well built” people; the men went about naked, although they may have used a loincloth, while the women used deerskin knee-length skirts. They wore long hair and facial and body paint [11, 12]. The Spaniards reported the Chichimecas as lazy, especially men, whose main duties were hunting and warring, while the rest of chores, including carrying the killed animals, were performed by women [7, 13].

Family ties were relaxed, and a woman would leave a man when she pleased [13]. It was said that men could have more than one woman if he had the means of maintaining them, and that they copulated “like animals,” indiscreetly and without privacy [11]. Babies were carried in a sort of basket, which was hung from trees while the mothers searched for food, protected at most by a deer skin [7].

Their social rules were simple, with weak leadership and band leaders effective only for warring [7, 12, 13]. According to the chroniclers, they had no clear religion, nor religious ceremonies [11]. Torquemada [7] indicated that they made self-sacrifices to stone and mud idols, bud Santa María [14], with firsthand knowledge, specifically denied it, while indicating that at most they would look to the sky and made exclamations to prevent being hit by lightning.

When antagonism between the Chichimecas and the Spanish became stronger, the Chichimecas fought the later naked and painted (if they had any cloths, they would remove them before fighting [14]), fearlessly, and with only bows and arrows as weapons, in whose use they were extremely dexterous [7, 12]. Their raids wreaked havoc among the Spanish soldiers and settlers for four decades, during which they caused significant losses [7, 12].

The Chichimecas were blamed for great savagery and said to be the worst homicides and robbers “in the whole world” [13: 211], and that “killing and robbing was their god” and “main objective” [11: 179]. They were said to carry bones to notch-tally the enemies killed [13], although there is no basis for such claim in earlier writings. The victims caught alive were treated with great fury: they were scalped, their tendons (to tie arrow points), long bones, and sometimes ribs were removed while the captive was alive, after which the “unfortunate” continued to be tortured until its death [14]. Chichimecas were accused also of cannibalism and said to eat the flesh and drink the blood of their enemies [7], letter by the cabildos in [9, 11, 12]; and, furthermore, that they killed children by tearing out their hearts and ate their bodies [12].

Most aspects of the daily life of the Chichimecas were poorly recorded at the time. Furthermore, living a semi-nomadic existence in seasonal grass and shrub huts or in caves did not leave material vestiges that allow the reconstruction of their lifestyle either, except for lithics, which are common [15]. This is not to say there are no pre-Hispanic vestiges in the area, there are many, but they were not created by Chichimecas but by Mesoamerican peoples in a northward Mesoamerican expansion. This expansion occurred from the year 500 before the Common Era (BCE) [16], grew to its maximum development around the year 900 of the Common Era (CE) [17, 18], and a complete collapse and retraction around 1200–1250 CE [19]. Actually, the confusing scenario described by Sahagún [4] of widespread Chichimeca movements in central México could involve withdrawal of Mesoamerican people from the northern extents into central Mexico.

The Chichimecas were a major source of concern for the Spanish colonizers as they opposed their northern expansion to the rich silver mines of Zacatecas, causing the most lengthy and costly war they faced in the new world [5]. The elusive nature of the Chichimecas, the fear that the Spaniards had of them and of the habitat they inhabited, and the Spanish inability to subdue them found their way into colonial culture, as is reflected by the many pages written about them. However, as we describe in the “Methods” section, few of these pages were derived from firsthand knowledge.

### The Guachichiles

The name Guachichil means red-headed and referred to the fact that the members of this group dyed their hair red or used small, pointed red caps (“like house finches” [14]). The Guachichiles were putatively an Uto-Aztec group that formed part of the non- or limited farming groups in northern Mexico [3, 8]. Their territory was crossed by the Spanish Camino de la Plata (the Silver Road, a section of the Camino Real de Tierra Adentro, the Royal Road of the Interior) between the current cities of Zacatecas and San Felipe (Figs. 1 and 2). This lead to them becoming major participants in the Chichimeca War. Their original lifestyle was poorly recorded because of several causes. Their settlements were diffuse and unknown to the Spaniards; the Spanish were focused on maintaining the operability of the Camino de la Plata, not on befriending the Guachichiles; and friars very seldom ventured beyond San Felipe. There is only one written record by a non-military; that of Fr. Guillermo de Santa María who interacted with the natives long after the onset of the conflicts and presumably after changes in their lifestyle had begun. Once the war was over, it was too late to rescue knowledge of their original lifestyle, and, anyhow, there was nobody doing it as the course of action was to force settlement and indoctrination. Modern study of Guachichiles was neglected, as Mexican archeology became predominantly centralist and defined on the basis of urbanism and agriculture [20].

Not much has been written specifically on the Guachichiles besides the accounts of Pedro de Ahumada [21] and Guillermo de Santa María [14] which are applicable mostly to them, but much of the information on Chichimecas more widely is probably applicable to them as well. Both Santa María [14] and Torquemada [7] considered the Guachichiles to be the worst offenders (to the Spanish interests) of all the Chichimeca peoples. They were also said to be the bravest and boldest [7]; although Santa María [14] indicated that to him, the bravest, more bellicose, treacherous and harmful were the Guamares, occupying areas of the current states of Guanajuato and nearby Michoacán (Fig. 2). The Spanish were not the only ones in regarding the Guachichiles with contempt but even a Zacatecan chief, Xiconaque, friendly to the Spanish, warned captain Pedro Almendez Chirinos, who was traveling northward from Comanja (near the current city of Lagos de Moreno) in 1530, that the Guachichiles were both treacherous and robbers [9].

The Guachichiles were divided into multiple groups, mostly associated with, and were named after specific leaders at the time of the conflicts, like the Macolias, Samúes, and Guaynamés [14]. The Maticoya were especially well known as, under the leadership of a certain Martinillo, they inflicted severe and well-publicized attacks on the Spanish [14], including the defeat of a party of 50 mounted and armed soldiers, “without leaving a single one to carry the news” ([7], vol 2: 428).

### Objectives

Almost 40 years have elapsed since the publication the Handbook of North American Indians [1, 2], and we still do not know much more about the Guachichiles. This is due to the fact that the few original accounts on them were analyzed time ago and no new original information has been found or generated. If we want to advance in our knowledge on the Guachichiles, we must rely on reviewed interpretations, more than on new factual information.

We here aim at providing a fresh interpretation of the relationship of the pre-contact Guachichiles with their environment to better understand their lifestyle by reviewing documentary materials from different approaches. We specifically hypothesized that people living in an environment as rich as the *tunales* did not need to resort to nomadism to obtain their necessary resources for survival and wellbeing. We searched for information that escaped previous researchers and recreated the pre-Columbian environment of the southernmost Guachichiles from an ecological perspective (Eric Mellink and Mónica E. Riojas-López), and a historical viewpoint (José Antonio Rivera-Villanueva) complemented with our ethnobiological acquaintance on the resources currently used by local inhabitants. Based on this, we re-valuate whether southernmost Guachichiles were nomads, as the Chichimecas collectively have been labeled, or whether those living in *tunales* could live year-round in this habitat. This required that we also determined the primary food sources and other consumable products of this group of Guachichiles based on the resources linked to the habitat that they inhabited. In doing so, we expect to provide a more accurate picture of the Guachichil lifestyle, to expand the vision of the diversity of habitat-mediated lifestyles of hunter-gatherers, and to re-valuate the neglected role of *tunales* in the regional biocultural heritage.

## Methods

Only four, perhaps five, documents providing firsthand information on the Chichimecas are known. The oldest document on the southern Chichimecas is a letter from 1543 directed by all the cabildos (administrative councils) of Nueva Galicia addressed to King Charles V, complaining about the native peoples in that region; it was reproduced in 1653 by Fr. Antonio Tello [9]. It is unclear if the endorsers had direct knowledge of the Chichimecas or were mostly wielding rumors. Secondly, the manuscript by Pedro de Ahumada, who fought the Guachichiles between the current cities of San Felipe and Zacatecas, and wrote in 1562 an account providing much information about these people [21]. Thirdly, the “Tratado de la Guerra de los Chichimecas” [14], written by Fr. Guillermo de Santa María, who spent 5 years in contact with the Guachichiles in San Felipe and San Francisco in the late 1560s and early 1570s. This work, written from 1575 to 1580 was for a long time erroneously credited to Gonzalo de las Casas, who had made a military incursion in the Guachichil territory in 1571. Fourthly, the detailed “Mapa de las Villas de San Miguel y San Felipe de los Chichimecas y el pueblo de San Francisco Chamacuero” (archived at the Real Academia de la Historia, Madrid) elaborated in 1579, and which provides pictorial information on the Chichimeca territory and aspects of the war. The map was likely not drawn by someone with firsthand knowledge of the area, but based on a written testimony, which has, regrettably, not been found. Fifth and lastly, Juan de Cárdenas, a physician from Guadalajara, wrote about some “curious” attributes of the Chichimecas in 1591 [11]. Cárdenas seems not to have personally met Chichimecas in their natural condition and appears to have credited rumors and myths, except in that he might have known first or secondhand about a few Chichimecas translocated to the city. Furthermore, his remarks are far from unbiased.

In addition, four of the most important chroniclers, three Franciscans and one Jesuit, of the Colonial period in Mexico wrote descriptions of the Chichimecas based on information provided by others, and perhaps based on widespread rumors. The best known is Fr. Bernardino de Sahagún, author of the “Historia general de las cosas de Nueva España,” also known as “Códice Florentino” [4]. Written shortly after the Spanish defeated the Aztec, this work was not published until the nineteenth century. After his arrival to Mexico, Sahagún never left the highlands of the central valleys, became a fluent Náhuatl speaker, and an expert on the Mexica culture, but his words on the sixteenth century Chichimecas must have come from indirect sources.

The second important chronicler, Fray Juan de Torquemada wrote a lengthy account on Mexico [7] based on codexes, paintings, manuscripts, oral tradition, and the work of other chroniclers, originally published in 1615. He assisted an unrepentant Chichimeca leader when he was hanged in Guadalajara, but this seems to be his only firsthand experience with these people. The third important chronicler is José Arlegui (1686–1750), a friar and inquisition functionary during the late seventeenth century, who spent much of his life as a missioner in Zacatecas and San Luis Potosí, and published the “Crónica de la provincia de NSPS Francisco de Zacatecas” [22] in 1737. This document included a number of remarks on native people, from Zacatecas to as far away as Nuevo León and the lands of the Rarámuri (the term that the Tarahumara, in the Sierra Madre Occidental in western Chihuahua, use for themselves). His descriptions were derived from personal observations as well as those of others, and possibly also widespread rumors and myths. In addition to the difficulty in clarifying what his own observations were, these were a century after the Guachichiles and the closely related Zacatecos had been subdued. The fourth important chronicler was Francisco Javier Alegre, a Jesuit scholar who was commissioned to write the history of Jesuits in the New Spain, after they were expulsed in 1767. He accomplished this task while in exile in Bologna, Italy, but his work was not published until 1841–1842 [13]. Although untimely to document the original characteristics of Chichimeca life, he might have had access to internal Jesuit documents that were not available to non-Jesuit chroniclers. Lastly, a royal chronicler in Spain, Antonio de Herrera y Tordesillas, wrote a description of the Americas [10] and, from 1601 to 1615, an extensive treatise on the history of Castillians in New Spain, based on information received in Spain [12]. Although he did not focus directly on native peoples, his work contains numerous notes on them and their way of life.

We carried out an exhaustive review of published literature, prioritizing the original sources, and performed extensive searches in the historical archives of the states of Jalisco, Zacatecas, Aguascalientes and San Luis Potosí, the historical archive of the Archbishopric of Guadalajara, and the Archivo General de la Nación, in Mexico City. From the pertinent accounts available, we defined a better circumscription of the habitat in which southern Guachichiles lived, and infer the resources available to them. In September and October 2016 and February 2017 we carried out field work to define a geographic explicit area of the Tunal Grande at the time of Spanish arrival, using the information in the primary sources as well as the presence of arborescent *nopalera* remnants, their relationship with topography and soil, and geographic names related to *nopaleras* or *tunales*. Upon this information, we compiled information of the wild animals and plants occurring in the *tunal* habitats, based on our personal knowledge of the plants and animals in the region, as well as current and historical ethnobiological information on use of wild resources in this and similar areas. Combining such different research approaches, we inferred the resources most likely included in the diet or otherwise used by the Guachichiles. The lists were integrated with species that are currently common or abundant in *tunal* habitat, large enough to be worth its hunting, and reasonably easy to hunt. Our study plan was an original work proposal as we have failed to find any other study reconstructing the ecology of a human group that was eradicated in the last hundreds of years without having been described carefully, and which did not leave behind physical vestiges related to their day-to-day living.

All translations from Spanish to English were performed by Eric Mellink and Mónica E. Riojas-López.

## Results

### The Chichimecas and their resources

According to the chroniclers, the lifestyle of the Chichimecas was rudimentary and deprived of riches [10]. Their lands, his Mexica informers told Fr. Bernardino de Sahagún, were poor, sterile, and without resources [4]. In these lands, they said, the Chichimecas subsisted mostly on hunting with bow and arrows. They deftly hunted deer, rabbits, jackrabbits, toads, lizard, snakes and “other poisonous animals,” and “sabandijas” (“creepy crawlies”), and, after their introduction, cattle, mules, and horses. Meat was consumed unwashed and half raw [7, 12]. Cárdenas [11: 179] claimed that animal meat was consumed only when human flesh, “their main sustenance,” was not available.

The other components of the Chichimeca diet were wild fruits: *tuna*, the fleshy fruit of *nopales* (prickly pear, *Opuntia* spp.), which allegedly supported Chichimecas most of the year, mesquite pods (*Prosopis laevigata*), *guamúchil* pods (*Pithecellobium dulce*), “dates” (surely *Yucca* spp.; see [23]), and unspecified “roots” [11, 12]. They also gathered hives [11], which would have been from the Mexican honey wasp (*Brachygastra mellifica*) and perhaps other congeneric species, whose honey was historically used and even sold at markets [24].

Wherever they occur, the *tunas* are not only eaten eagerly but throughout Aridoamerica they were, and still are, used to prepare alcoholic beverages. The Chichimecas were not an exception and prepared such a drink (*colonche*, as it is known nowadays, which is still prepared in the Tunal Grande area). Although alcoholic beverages made from roots by the Chichimecas were mentioned [12], no further information allows for its verification. Not only did the Chichimecas indulge in alcoholic beverages, but they were reported to also use peyote (*Lophophora williamsii*), which helped them maintain high spirits, fearlessness, and lack of hunger and thirst during their wartime skirmishes [4].

A critical element for human survival is water. Most chronicler did not mention anything about it, and the only remark was by Cárdenas [11]. He asserted that Chichimecas could spend months, or even their entire life without drinking water; but that if water was present, they could drink more than a [thirsty] horse.

Despite the Spanish contempt for Chichimeca foods and lifestyle, they had notoriously good health, but when they were taken to settled Spanish habitation and fed colonial food, they became frail and “a picture of diseases,” and a small pain or diarrhea were enough to cause their death [11: 180]. Cárdenas [11] attributed this to the change in diet, the lack of exercise, and the lack of fresh air.

### The southernmost Guachichiles

**Fig. 3:**
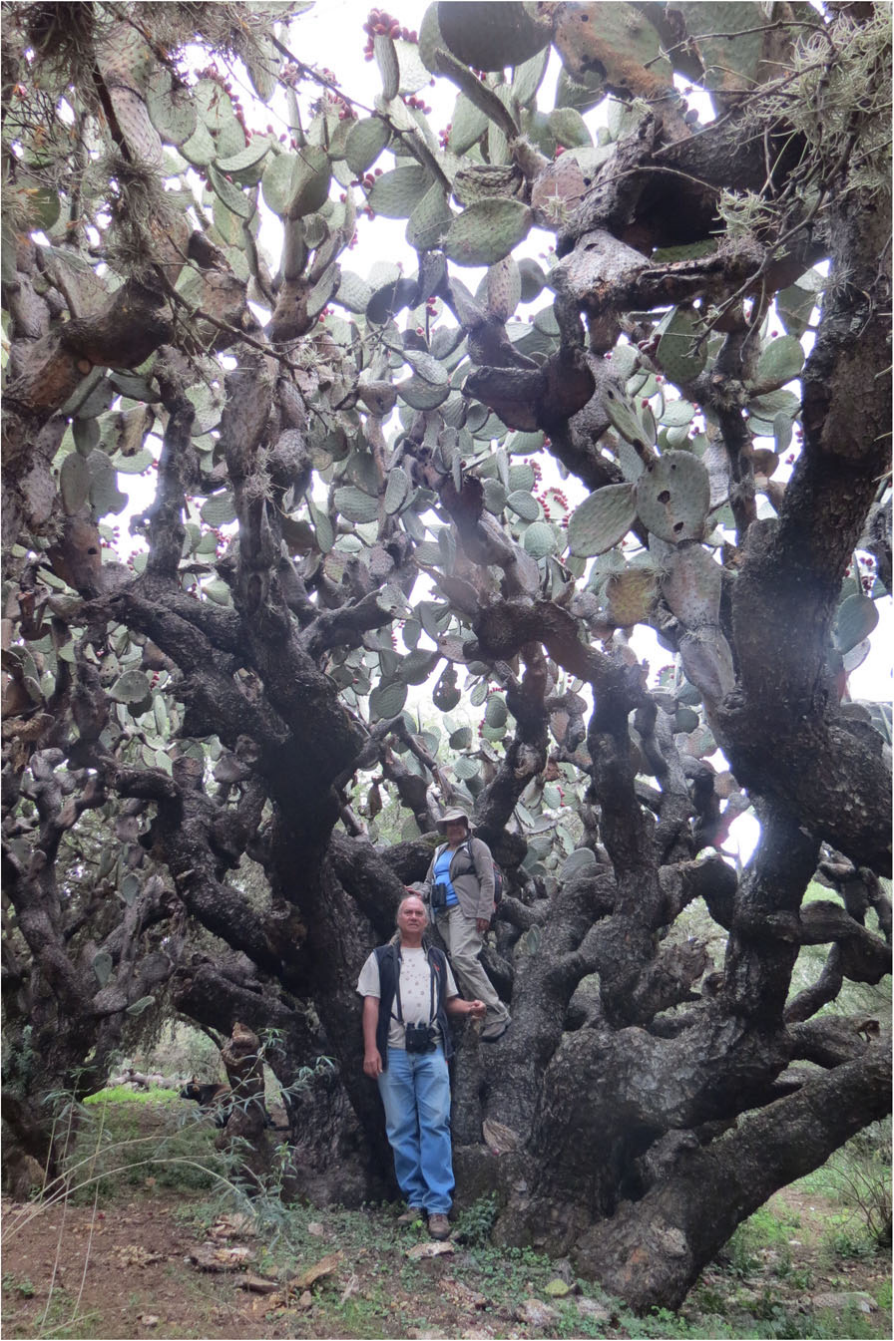
Remains of a magnificent *Tunal* near Charcas Viejas, San Luis Potosí. In image, Mónica E. Riojas-López and Eric Mellink. Photograph by José Antonio Rivera-Villanueva. September 2016

**Fig. 4:**
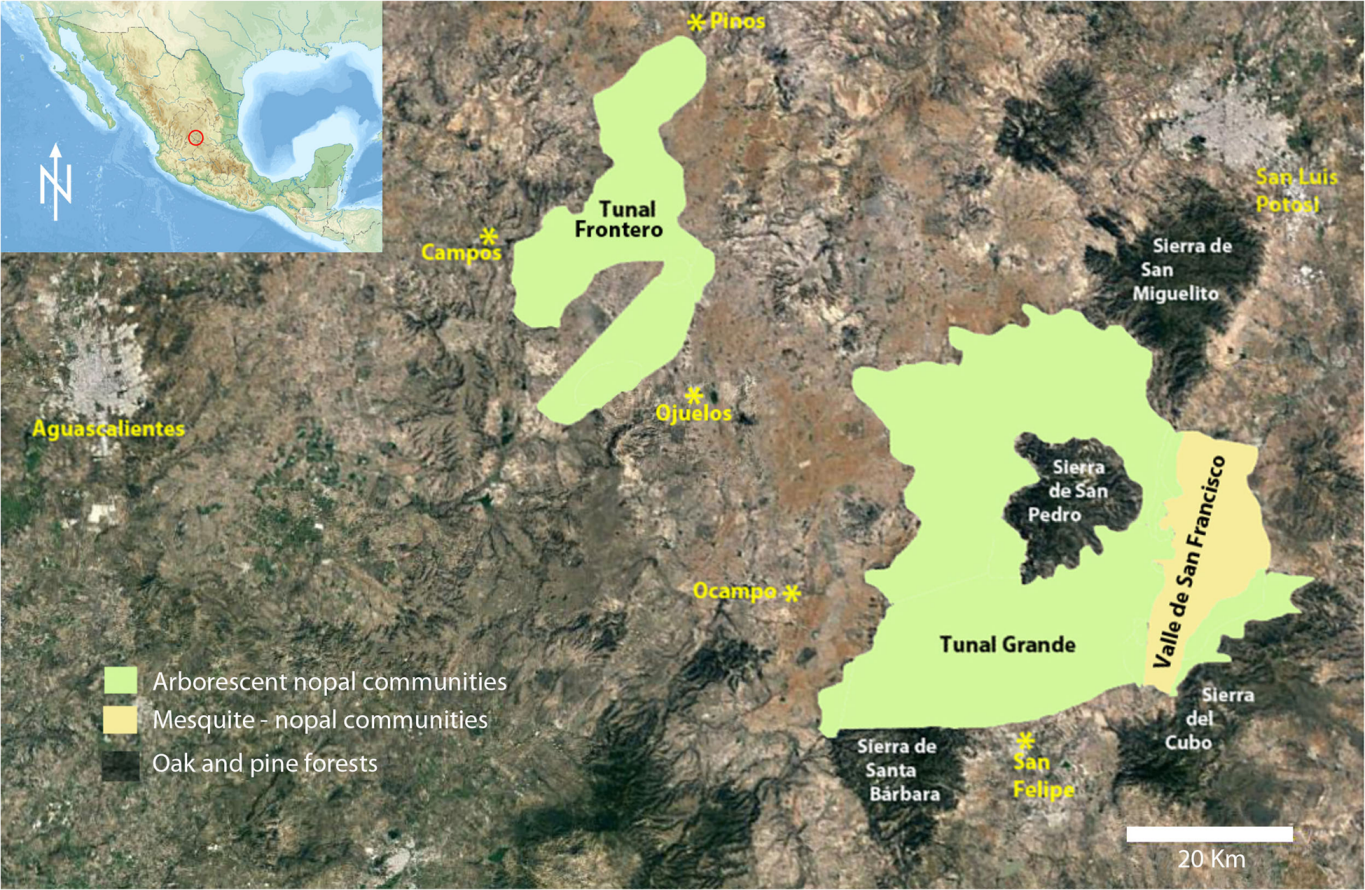
Historical minimal extension of *Tunal Grande* and *Tunal Frontero*. Their extent was delimited based on Ahumada [21] and field work by the authors. Base map: Google Earth, ©2014 DigtalGlobe

All the descriptions about the Chichimecas and the Guachichiles have overlooked one major, and certainly not trivial, difference between the southern fraction of Guachichiles and the rest of Chichimecas: the habitat they occupied. While the other occupied grasslands, open shrublands, and perhaps oak or pine-oak woodlands, the southern Guachichiles had taken possession of the *tunales* [25]. These were literally forests of arborescent forms of *nopal* (*Opuntia streptacantha*, *O. lasiacantha*, and *O. chavena*; Fig. 3), sometimes mixed with mesquites (*Prosopis laevigata*) and wild *maguey* (*Agave salmiana* spp. *crassispina*; not to be confused with the domestic maguey, *Agave salmiana* var. *salmiana*, brought in from central Mexico by the Tlaxcaltecas later). One such *tunal* was said to measure over 200 km in length [12]. Some spots within *tunales* were so dense that it prevented the Spanish from fighting on horseback [21]. *Tunales* were distributed from about San Felipe to beyond San Luis Potosí to the north and beyond Zacatecas to the northwest. No map exists of their pre-contact extent or even early twentieth century extent, but Ahumada’s account of his search for Guachichiles in two *tunales* [21] allowed us to reconstruct their minimal extent (Fig. 4; [26]). Our interpretation of Guachichil lifestyle is based on reports mostly from the southeastern one of these, named Tunal Grande by Ahumada [20].

In the *tunales*, the Guachichiles “…*tienen mucha cantidad de tuna blanca e colorada de diversos géneros*…” (have large amounts of white and red *tuna* of different types [21]). *Tunas* were produced from May to October, and mesquite pods from October to December, a little less than Pedro de Ahumada’s contention of 8 and 4 months, respectively [21], providing for a lengthy season of resources. Moreover, Guachichiles made cakes from ground mesquite pods, which they used during the rest of the year [14]. In addition to *tunas* and mesquite pods, *tunales* provided plenty of other plant resources, and at least other seven “plant” products were confirmedly or very likely important components of their diet (Table 1). Some of them continued to be used in the area afterwards, like flower stalks of agaves (cooked), which were much appreciated in San Luis Potosí in the early nineteenth century [27], and, along with *tunas* and *cabuches* (barrel cactus flowers) are highly prized today. Yucca flowers have also been indicated as part of the diet of the hunter-gatherers of northern Mexico [28]. The consumption of pinyon nuts from the Mexican nut pine by Guachichiles was not recorded, but they needed to make only a short foraging trip to the nearby mountains to gather them, especially in mast years when the reward would have been high. The *guamúchil*, whose pods are still highly prized and were consumed by other Chichimeca groups [11], does not occur in the area occupied by the Guachichiles.

**Table 1:** Plants and fungi most likely to have been used by the southernmost Guachichiles

Resource	Species	Notes
Tunas	*Opuntia* spp.	Eaten fresh
Mesquite pods	*Prosopis laevigata*	To prepare a bread
Dates	*Yucca decipiens* and *Y. filifera*	As fruits, eaten fresh
Roots similar to potatoes	S*olanum cardiophyllum* and *S. ehrenbergii*	*Solanum* spp. are still sold locally for food.
Roots similar to potatoes	*Phaseolus coccineus*	Root noodles possibly eaten.
*Maguey*	*Agave salmiana* spp. *crassispina*	Cooked roots, leaves, and flower stalks eaten.
*Biznaga* (barrel cactus)	*Ferocactus histrix*	Flowers (currently known as *cabuches*, a highly appreciated delicacy) and fruits eaten.
Pincushion cacti	*Mamillaria* spp.	Fruits eaten.
*Garambullo* (bilberry cactus)	*Myrtillocactus geometrizans*	Fruits eaten.
Mexican nut pine	*Pinus cembroides*	Pinyons probably eaten.
Peyote	*Lophophora williamsii*	Likely to have been obtained by trade for use as hallucinogenic
Beehive cactus	*Coryphanta* spp.	Possibly used as hallucinogenic
Psychedelic mushroom	*Psilocybe* spp.	Possibly used as hallucinogenic

While living in the *tunales*, they would not have to leave the safety of this habitat to hunt at least 18 species of birds, mammals, reptiles, and amphibians (Table 2). Animal food obtained within the *tunales* would have been augmented with at least six species that could be hunted at the borders between them and grasslands or shrublands, or in the more open patches of *tunal* (Table 3). All species included in Tables 2 and 3 are permanent residents in the habitats indicated, except most sparrows which are migratory but are present in the area for at least 6 months. Grubs would not have been overlooked as dietary items, including the larvae of *Melitara nephelepasa*, a snout moth. This grub, which can grow to about 2 cm long, develops inside *nopal* pads, from which it is sometimes collected by local peasants and eaten fried. It is likely that several other grubs were consumed as well, but paucity of information on invertebrates in arboreal *nopalera*s or in any other natural habitat in the region prevents us from explore this further. Several of the species of animals likely to have been used by the Guachichiles were still used a few decades ago or are still used today (Tables 2 and 3; [29]; Eric Mellink and Mónica E. Riojas-López unpub. obs.).

Guachichiles, like other Chichimecas, were fond of drunkenness (but then, as Guillermo de Santa María [14] remarked “no nation has been found that is content with drinking only water”). Their major alcoholic beverage was *colonche*, fermented *tuna* juice, but they were said to also prepare *vino mezcal*, from the native maguey, and an alcoholic beverage prepared with mesquite pods [14]. They were so fond of colonche that they allegedly would drink until “unconsciousness every third day” [13].

**Table 2:** Animal species most likely used as foods, procured within *tunales* by the southernmost Guachichiles

Common name^a^	Scientific name	Current use^b^
Birds (individuals of all ages and eggs)		
Mourning dove	*Zenaida macroura*	As food
White-winged dove	*Zenaida asiatica*	As food
Canyon towhee	*Melozone fusca*	None known
Curve-billed thrasher	*Toxostoma curvirostre*	As cage bird
Northern mockingbird	*Mimus polyglottus*	As cage bird
Cactus wren	*Campylorhynchus brunneicapillus*	None known
Finches and sparrows	Passerellidae, Fringilidae	Some species used as cage birds
Small mammals		
Packrat	*Neotoma leucodon*	As food, highly appreciated
Mexican spiny pocket mouse	*Liomys irroratus*	None known
Deer mice	*Peromyscus melanophry*, but likely also smaller species	None known
Rock squirrel	*Otospermophilus variegatus*	As food, occasional
Cottontail rabbit	*Silvilagus audubonii*	As food
Large mammals		
Collared peccary	*Pecari tajacu*	None, locally extirpated
Reptiles		
Rattlesnakes	*Crotalus* spp.	Used as a cancer remedy
Gopher snake	*Pituophis deppei*	None known
Scaled lizards	*Sceloporus* spp.	None known
Mud turtles	*Kinosternon integrum*, *K. hirtipes*	Blood used as a remedy
Invertebrates		
Grubs	The larvae of at least *Melitara nephelepasa*; possibly other species as well	Eaten occasionally
Mexican honey wasp (its honey)	Possibly *Brachygastra mellifica* and perhaps other congeneric species	None known, but likely to be still consumed

^a^Species within groups are ranked according to their probable importance, based on abundance in these habitats, size, and easiness of hunting. All species except the sparrows were permanent residents in the *tunales* (source: EM and MERL, pers. obs.)

^b^Current use derived from personal acquaintance of the authors and [26]

**Table 3:** Animal species most likely hunted at borders or in open *tunal* patches by southernmost Guachichiles

Common name^a^	Scientific name	Current use
Birds (individuals of all ages and eggs)		
Quail	*Callipepla squamata* and *Colinus virginianus*	As food
Small mammals		
Jackrabbits	*Lepus californicus* and *L. callotis*	As food
Cotton rats	*Sigmodon* spp.	None known
Large mammals		
White-tailed deer	*Odocoileus virginianus*	As food, but locally very reduced populations.
Pronghorn antelope	*Antilocapra americana*	Extirpated
Amphibians		
Leopard frogs	*Lithobates montezumae* and *L. neovolcanica*	None known

^a^Species within groups are ranked according to their probable importance, based on abundance in these habitats, size, and easiness of hunting. All species were permanent residents in the area (source: Eric Mellink and Mónica E. Riojas-López, pers. obs.)

Alcoholic beverages could have been complemented with hallucinogenic plants. The peyote (*Lophophora williamsii*), which seems to have been well used by other Chichimecas, is not found within the *tunal* area. It is found north of it, albeit not far away (in Venado, for example; José Antonio Rivera-Villanueva unpub. obs.), and could have been easily collected on short foraging trips from the northernmost *tunal* occupants, or traded from extra-*tunal* Guachichiles, and then traded throughout the *tunales*. Regardless of this, several *Coryphanta* spp. species have psychoactive phenylethylamines [30] and, potentially to their joy, *C. ottonis* and *C. cornifera* occur in the *tunal* areas (they have probably not been screened for such alkaloids yet). In addition, hallucinogenic fungi (*Psilocybe* spp.) occur in the area and would have flourished on peccary dung.

## Discussion

Contrary to what was considered at the time, *tunales* were rich in resources [3]. These exuberant habitats (Fig. 3) provided, at least, 10 plant foods (Table 1), 17 edible vertebrates, in addition to bird eggs, grubs, and honey (Table 2), which could be complemented by hunting along the edges of the *tunal* with grasslands and shrublands or in more open patches of *tunal* (Table 3). In addition to food, they prepared at least three alcoholic beverages and had access to hallucinogenic sources (Table 1). Not only were the flowers of one yucca species (*Yucca filifera*) potential food, but also those of *maguey*, both of which are consumed by peasants to this day. However, whether the Guachichiles ate them cannot be established. These flowers contain high levels of saponins [31], and to make them eatable, they are cooked in boiling water. As no records of earthenware capable of withstanding boiling among the Guachichiles exist, it seems unlikely that they could consume these flowers. Finally, stands of oak trees (*Quercus* spp.) were easily accessible for *tunal* inhabitants, but oaks native to the area produce only inedible acorns, as far as we know.

The major plant foods were so important that their chronology of fruit production drove the annual cycle in the life of these Guachichiles, who would resort mostly to hunting from January to April (sensu [32]). However, against Griffen’s remark [32], the Guachichiles hunted not only when fruits were unavailable, but also on a daily basis [14].

Although at the time, the Chichimeca diet was considered uncivilized and unhealthy, the truth was contrary, as demonstrated by those that were taken to the city, changed to a colonial diet, and soon began to fall ill and die, as Cárdenas [11] reported. This observation was merely anecdotal, but superiority of hunter-gatherer diets has been documented more rigorously for other peoples, like the Australian Aboriginal hunter-gatherers [33]. In the case of diet quality too, the southern Guachichiles would have enjoyed a superior diet to that of other Chichimeca groups; one that included a rich combination of plant and animal products, and can be presumed to have been rich in protein, energy, and vitamins. Such a diet would have provided them with a complete, reasonably well balanced diet.

The claim that the Chichimecas could live without water is completely unfounded, as humans are obliged to consume it [34], and water in arid lands can be obtained from different sources. One is the consumption of *tunas*, and these actually provided much of the water in the Chichimeca diet [12]. Blood is also a source of water [34], and Chichimecas could have plenty of it from the animals they hunted (turtle blood is still consumed occasionally in the San Luis Potosí arid region, as a remedy for disease [29]). As a last resource, water could be obtained from succulent cacti stems and pads. However, as happens with other human groups inhabiting arid and semiarid lands, the most important way in which Chichimecas satisfied their water needs must have been their precise knowledge of the location of waterholes and other water sources. Many colonial-time Spanish documents attest that these were abundant and found through the entire region. The claim that they would drink water as much as a thirsty horse if available is untenable.

Cárdenas [11] statement that Chichimecas consumed animal meat only when human flesh was not available is clearly unrealistic and was grounded in the many myths about these peoples. This does not discard completely the likelihood of them performing occasional cannibalism, which was very likely done given its frequency among human groups [35]. It would have involved at least sacrificial cannibalism, but perhaps also political and, or mortuary cannibalism (sensu [35]).

**Fig. 5:**
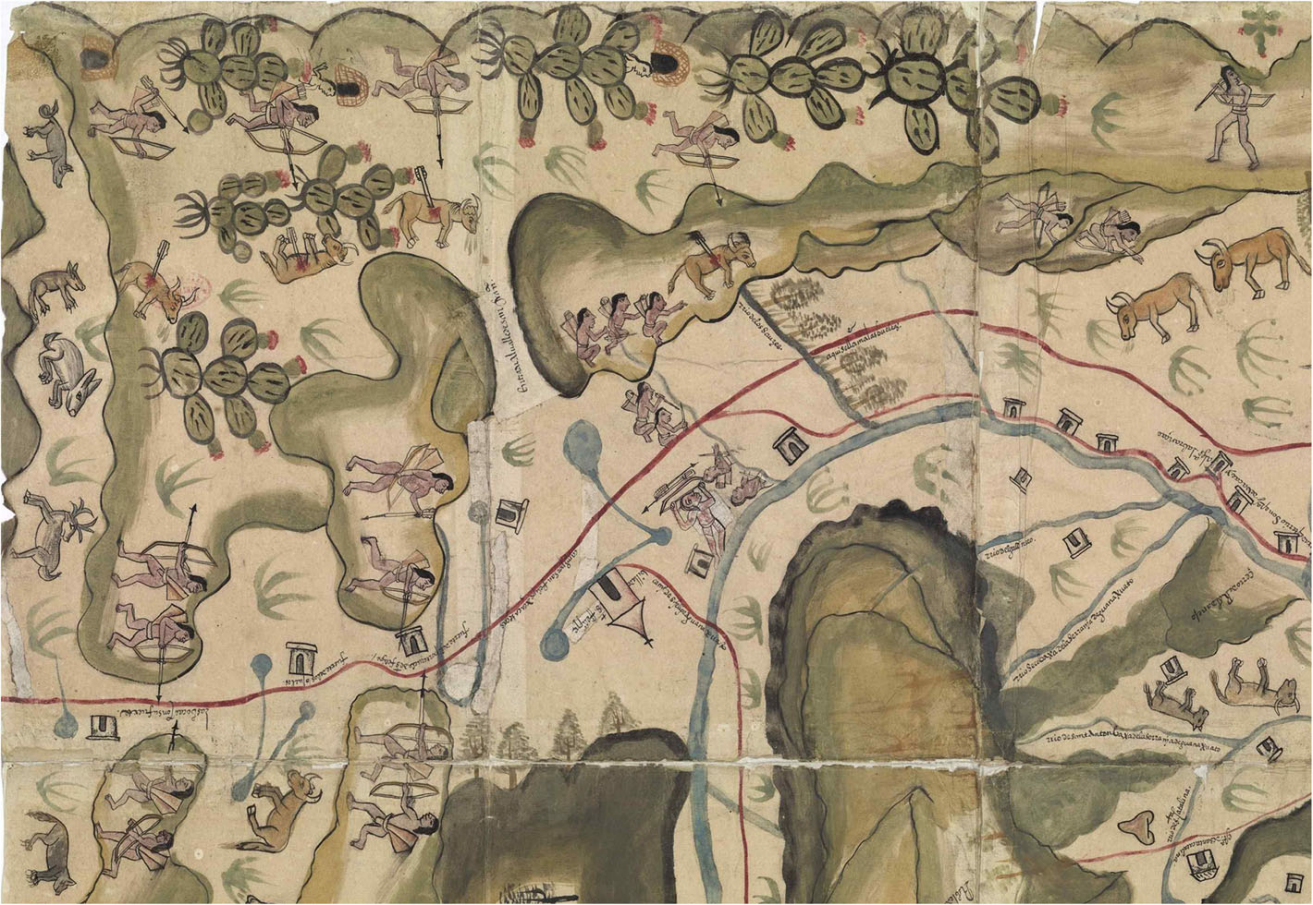
Excerpt of the 1579 “Mapa de las Villas de San Miguel y San Felipe de los Chichimecas y el pueblo de San Francisco Chamacuero” depicting *tunal* habitat and Guachichil huts.

People that lived in the *tunales* had a privileged life, and they could afford to remain stationary for long periods of time, as long as they did not deplete the *tunas* and wildlife from their surroundings. In contrast to the reported nomadism of the Chichimecas in general, the Guachichiles in Tunal Grande did not have to be always on the move, and they might have been settled in huts, as was depicted in the 1579 “Mapa of the Villas de San Miguel y San Felipe de los Chichimecas y el pueblo de San Francisco Chamacuero” (Fig. 5; the names of these villages changed to San Miguel El Grande and, later, San Miguel de Allende; San Felipe Torres Mochas; and Comonfort, respectively, all in the current state of Guanajuato). Even outside the fruiting season, game within the *tunales* was probably enough to sustain the Guachichiles, as long as they had taken care to prevent other groups from hunting there. Thus, although the Chichimecas are considered fully nomadic [36], the Guachichiles occupying the *tunales* do not fit into this category, and should not be considered as such. They seem to defy a clear classification, as they would be closer to Murdock’s [36] “neighborhoods of dispersed family homesteads,” but with neighborhoods probably often relocated in response to food availability. Those in Tunal Grande would be occupying an area roughly about 400 km^2^ (Fig. 4).

South of the Guachichil territory, towards where now is the city of Lagos de Moreno, the Guamares, another Chichimeca group, had abundant subgroups that subsisted by hunting rabbits, jackrabbits, and deer on the move, and slept wherever the night caught them [9]. The Guachichiles in *tunales*, arguably better fed and in better body condition would be wise to deter open-habitat neighbors from hunting on their grounds. This is strongly supported by the Chichimecas’ frequent bloody intra-group conflicts [7, 14].

To the north of the Tunal Grande and nearby *tunales*, there were allegedly Guachichil people all the way to current day Mazapil, in northern Zacatecas, Saltillo, in Coahuila, and Monterrey, in Nuevo León ([37]; José Antonio Rivera-Villanueva, unpub. obs.). If this was indeed so, we can hypothesize that the Guachichiles in the *tunales* excluded also those other, more northerly Guachichiles from their foraging and hunting grounds. This would be reflected in infra-group divisions akin to those of other indigenous groups of North America which had clearly defined sub-groups, like the O’odham (Tohono, Akimel, and Hia C-ed O’odham [38]), and Comcáac (San Esteban and Tiburón island groups in Sonora [39]). Thus, in the case of the Guachichiles, based on the fact of a certain fraction of them occupying the rich *tunales*, the reported presence of Guachichiles as a far north as Saltillo, and their reported territorial defense, we hypothesize that at least two different habitat-defined groups of Guachichiles existed. One would be the *tunal* peoples; another, north of the *tunales*, would be the grassland-shrublands Guachichiles, or perhaps a grassland and a different shrubland group.

What impacts the Guachichiles had on the environment cannot be precisely assessed, but they most likely had some. As happens sometimes nowadays with the hunting of packrats, they could have reduced local populations of some prey species by hunting, and those of some birds by scaring adults from the nests and taking the eggs, but none of these would have had a permanent effect on the biological integrity of the *tunales*. Indigenous groups in North America’s Great Plains have influenced the environment through habitat burning for hunting or for war [40]. Unlike them, there is no record that the southern Guachichiles, nor any other Chichimeca group, burned the habitat on purpose, perhaps because overall there was not enough ground-level fuel to sustain an effective fire. Occasional wildfires could have been caused by accident, especially in autumn if it had been a rainy summer and dry herbage abounded, but it seems not likely that this had a major impact on the local habitats.

Janzen [41] argued that prehistoric human occupants of the region could have established orchards. We find this far-fetched. Even if the Mesoamericans in the northern Epiclasic cities had planted orchards, their abandonment occurred over seven centuries ago, time enough for conditions to have reverted to natural. On their part, Chichimecas, including the Guachichiles, did not develop any form of agriculture. Rather, the Guachichiles might have influenced the species composition in certain areas just by defecating the seeds of their preferred tuna species around camps (as is the case nowadays with the *tuna cardona*, *O. streptacantha*).

*Tunales*, for the most part, have been neglected as a source of richness and have been viewed rather as obstacles to agriculture and progress. Neglect of this habitat began with the Spaniards, who feared it and associated it with the “primitive, poor” and antagonist Guachichiles. They were neither compatible with the ranching nor farming systems implanted by the Spaniards, and with their later development. As a result, this type of vegetation has not been appreciated and protected, and has been destroyed slowly until their almost extirpation from the region nowadays. However, as our analysis has shown, they were rich habitats capable of supporting native human groups year-round. They played a critical role in the pre-Columbian arrangement of people in the region and in the resistance of southern Guachichiles to the Spanish. In congruence, *Tunales* should be recognized as a fundamental component of the regional biocultural heritage.

Carrying out the work that we report in this article showed that tackling the multidisciplinary issue of people’s interaction with the environment by a team conformed by social and biological scientists is profitable and can generate new, fresh insights, in addition to be very stimulant and formative for the participants. Our article points at the importance for scientists in the natural resources to adopt a wider temporal scope including historic sources and even archival work, as these can provide insights into the history and current status and use of such resources by the local population. At the same time, it exhibits the depth that social scientists can obtain to understand the changes in the region of their interest by including knowledge about landscapes, habitats, and wild flora and fauna.

## Conclusions

1. Food resources were abundant and varied for the southern Guachichiles, those living in *tunal* habitats, and such habitats were absolutely not the inhospitable places indicated by Fr. Bernardino de Sahagún’s informants. Such richness reflect in that many of the uses that Guachichiles made of *tunal* resources are still practiced nowadays.
2. Due to abundant food resources, in all likelihood, the southern Guachichiles were not nomadic, but lived in moving neighborhoods of dispersed family homesteads.
3. There might have been at least two or three different Guachichil groups: The *tunal* Guachichiles, and the grassland-shrubland Guachichiles; or even, perhaps, grassland Guachichiles and shrubland Guachichiles. The prior would have prevented the other groups, as well as the Cascanes to the south, from hunting in *tunal* habitats.
4. The majestic, spiny, and closed arborescent *nopalera*s were the Mexican “black forest” for the Spanish, imposing great fear upon them, which, along with Guachichil “savagery” can explain how the later resisted being subdued for much longer than other native peoples.
5. *Tunales*, despite having been neglected for a long time, should be recognized as a fundamental component of the regional biocultural heritage.
